# Exploring the Potential of Aptamers in Targeting Neuroinflammation and Neurodegenerative Disorders: Opportunities and Challenges

**DOI:** 10.3390/ijms241411780

**Published:** 2023-07-22

**Authors:** Anna Hau-Yee Kong, Aston Jiaxi Wu, Olivia Ka-Yi Ho, Maggie Ming-Ki Leung, Alexis Shiying Huang, Yuanyuan Yu, Ge Zhang, Aiping Lyu, Min Li, King-Ho Cheung

**Affiliations:** 1 Teaching and Research Division, School of Chinese Medicine, Hong Kong Baptist University, Hong Kong SAR, China; 21481989@life.hkbu.edu.hk (A.H.-Y.K.); astonwu@hkbu.edu.hk (A.J.W.); oliviaho@hkbu.edu.hk (O.K.-Y.H.); 19225547@life.hkbu.edu.hk (M.M.-K.L.); 17482429@life.hkbu.edu.hk (A.S.H.); limin@hkbu.edu.hk (M.L.); 2 Law Sau Fai Institute for Advancing Translational Medicine in Bone & Joint Diseases, School of Chinese Medicine, Hong Kong Baptist University, Hong Kong SAR, China; yuyuanyuan@hkbu.edu.hk (Y.Y.); zhangge@hkbu.edu.hk (G.Z.); aipinglu@hkbu.edu.hk (A.L.); 3 Guangdong-Hong Kong-Macao Greater Bay Area International Research Platform for Aptamer-Based Translational Medicine and Drug Discovery, Hong Kong SAR, China

**Keywords:** aptamer, neuroinflammation, neurodegeneration, alzheimer’s disease, parkinson’s disease, microglia

## Abstract

Neuroinflammation is the precursor for several neurodegenerative diseases (NDDs), such as Alzheimer’s disease (AD), Parkinson’s disease (PD), and multiple sclerosis (MS). Targeting neuroinflammation has emerged as a promising strategy to address a wide range of CNS pathologies. These NDDs still present significant challenges in terms of limited and ineffective diagnosis and treatment options, driving the need to explore innovative and novel therapeutic alternatives. Aptamers are single-stranded nucleic acids that offer the potential for addressing these challenges through diagnostic and therapeutic applications. In this review, we summarize diagnostic and therapeutic aptamers for inflammatory biomolecules, as well as the inflammatory cells in NDDs. We also discussed the potential of short nucleotides for Aptamer-Based Targeted Brain Delivery through their unique features and modifications, as well as their ability to penetrate the blood-brain barrier. Moreover, the unprecedented opportunities and substantial challenges of using aptamers as therapeutic agents, such as drug efficacy, safety considerations, and pharmacokinetics, are also discussed. Taken together, this review assesses the potential of aptamers as a pioneering approach for target delivery to the CNS and the treatment of neuroinflammation and NDDs.

## 1. Introduction

With rapidly ageing populations, the increasing cases of neurodegenerative diseases (NDD) and their socioeconomic impact have drawn great interest from researchers worldwide. However, there remains a lack of effective interventions to reverse the progressive destruction of neurons or a comprehensive understanding of the underlying mechanisms.

Most therapeutic interventions in NDDs focus mainly on pathological features such as abnormal amyloid and tau-tangle accumulation, mitochondrial dysfunction or neurotransmitter imbalances [1,2]. These approaches might ameliorate neurotoxicity from abnormal metabolites and temporarily slow disease progression, but misfolded proteins, mitochondrial dysfunction or neurotransmitter imbalance may not be the sole contributing factors to NDDs. Indeed, other factors, including inflammation, oxidative stress, and genetic predisposition, may also play a primary role in disease development and progression [3]. Additionally, targeting misfolded proteins may not be sufficient to treat NDDs, as they can also exist in a functionally folded form in healthy individuals [4]. For instance, the presence of amyloid-β in the brain images of cognitively healthy older individuals has often been criticized as a weakness of the approach [5,6,7], while the observation of Lewy bodies in the brains of older individuals without any PD symptoms has also been noted [8,9]. The accumulation of intracellular amyloid-β and Lewy body pathology in the corresponding brain regions is recognized as a hallmark of AD and PD, respectively. Substantial evidence is growing that α-synuclein plays a key role as a mediator in inflammation and immune responses, is released from inflamed neurons and may exert a self-amplifying proinflammatory reaction [10]. Chronic neuroinflammation is a common feature among NDDs. Therefore, targeting the molecular pathways and immune cells involved in prolonged inflammation is gaining momentum as an auxiliary disease-modifying strategy for treating neurological disorders, supported by increasing evidence from epidemiological studies, neuroimaging, and genetics research [11].

Growing evidence has revealed the interdependent relationship between the nervous system and the immune system during the pathologies of neurological disorders, while the immune system is traditionally considered to be primarily regulated by its own autonomous mechanisms [12,13,14]. The concept of “inflamm-ageing” was proposed to describe a persistent state of inflammation that is present in many age-related diseases [15]. The immune response in the central nervous system (CNS) involves a combination of local and peripheral components, encompassing the brain, spinal cord, optic nerve, and retina [16].

Neuroinflammation acts as a defence mechanism to protect the brain from various pathogens, tissue injuries, toxins or other forms of stress in the CNS through the production of different signalling molecules, including proinflammatory cytokines such as IL-1β, IL-6, and TNF-α, as well as chemokines and other molecules such as reactive oxygen species (ROS) and nitric oxide (NO) [17,18,19]. This inflammatory response is initially favourable, as it promotes tissue repair and clearance of cellular debris [14,20]. However, uncontrolled and persistent inflammatory responses can be devastating and contribute to secondary injuries such as synaptic dysfunction and progressive destruction of neuronal cells [18,21,22]. These factors lead to cognitive impairment, movement disorders and sensory decline in the development of NDDs. Such prolonged inflammatory stimulation can result from endogenous factors such as genetic predispositions and ageing, as well as environmental factors such as oxidative stress, imbalanced lifestyle, infection, trauma, and toxins [13,23,24]. More importantly, severe neuroinflammation disrupts the integrity of the blood–brain barrier (BBB), which enhances the permeability and facilitates the recruitment of peripheral innate immune cells into the brain [18,25]. These circumstances trigger an inescapable cycle that further exacerbates the inflammatory response and accelerates the progression of NDDs.

Although molecular pathophysiology research has revealed differences among NDDs in terms of the pathological regions and pathogenesis, overactivated neuroinflammation is widely recognized as a pivotal factor in driving disease progression and pathogenesis among NDDs [26]. With the remarkable versatility of a new class of drugs known as small nucleic acid aptamers, an increasing number of them have shown early success in drug discovery research, and 14 of them have gradually entered clinical trials over the past few decades [27]. The application of oligonucleotide-based therapies in neuroscience has rapidly emerged in recent years. This innovative therapeutic approach sheds new light on the possibility of treating NDDs that were previously considered nonpreventable and incurable.

In the present review, we provide a comprehensive overview of the current understanding of neuroinflammation in the context of NDDs, including its underlying mechanisms and current treatment approaches. Furthermore, we discuss the feasibility of using innovative drugs, aptamers, as a diagnostic and therapeutic intervention from a neuroinflammatory perspective by exploiting their unique opportunities and challenges.

## 2. Neuroinflammation

### 2.1. Role of Microglia

Microglia, as resident myeloid macrophages in the brain, are primarily involved in neuroinflammation. The role of microglia in regulating homeostasis, tissue repair and immune surveillance by remodelling the extracellular matrix is critical [17,28]. Microglia are found in both grey and white matter and constitute approximately 0.5–16.6% of the total human CNS cell population [29,30]. Primitive microglia originate from yolk sac progenitors and are subsequently transported to the developing brain during embryonic development [31]. Although the subsequent maintenance of the microglial population is quiescent and relies on self-renewal capacity, microglial subpopulations can undergo site-specific clonal expansion to respond to local perturbations in disease states [32,33].

Microglial senescence, characterized by dysfunctional and activated microglia, contributes to the development of neuroinflammation. Senescent microglia are recognized as culprits of neuroinflammation due to their being hyperactivation-prone in the aged brain, causing phagocytosis of synapses and potentially inappropriate synaptic pruning, as well as a dysregulated inflammatory response [34]. Given their physiological and pathological features, microglia are increasingly recognized as a particularly vulnerable target for ageing and neurodegeneration, far more so than previously believed.

### 2.2. Activation of Microglia

Microglial activation is considered the first sign of neuroinflammation [22,35]. In the pathologies of NDDs, the initial activation is triggered by the detection of extracellular or intracellular pathogen-associated molecular patterns (PAMPs) or damage-associated molecular patterns (DAMPs) through pattern recognition receptors (PRRs), such as the best-known Toll-like receptors (TLRs) and nuclear oligomerization domain-like receptors (NLRs) [36]. Myeloid differentiation factor 88 (MyD88) is a key adaptor protein involved in the signalling pathways downstream of TLRs [37]. MYD88-dependent signalling is initiated by ligand binding to TLRs expressed on the cell surface or intracellularly and is typically associated with proinflammatory responses [38]. DAMPs are endogenous molecules that signify cellular distress and are released from damaged or dying neurons, including the aggregates of the misfolded proteins amyloid-β, tau protein, and α-synuclein [39,40,41].

In response to these stimuli, activated microglia and astrocytes can initiate the synthesis of intracellular NLRP3 inflammasome proteins by activating the NF-κB pathway and inhibiting the AMPK pathway [42]. The NF-κB signalling pathway is the key player in the production of proinflammatory cytokines associated with the senescence-associated secretory phenotype. NF-κB is a transcription factor that induces the gene expression of several proinflammatory cytokines and activates NLRP3 during inflammation [43]. Oligomerization of the NLRP3 protein can then lead to the formation of a functional inflammasome complex, which can trigger the production and release of proinflammatory cytokines such as interleukin-1β (IL-1β), IL-6 and IL-12, thereby recruiting other immune cells to lesion areas and eliminating potentially damaging pathogens or tissue injury [44]. The release of proinflammatory cytokines can trigger the overactivation of protein kinases on neuronal receptors, such as those found in hippocampal and substantia nigra neurons, contributing to the formation of tau and α-synuclein polymers, respectively [45]. Interestingly, this series of pathological events of glial activation can be applied in several different NDDs.

### 2.3. Morphological Plasticity

**Figure 1:**
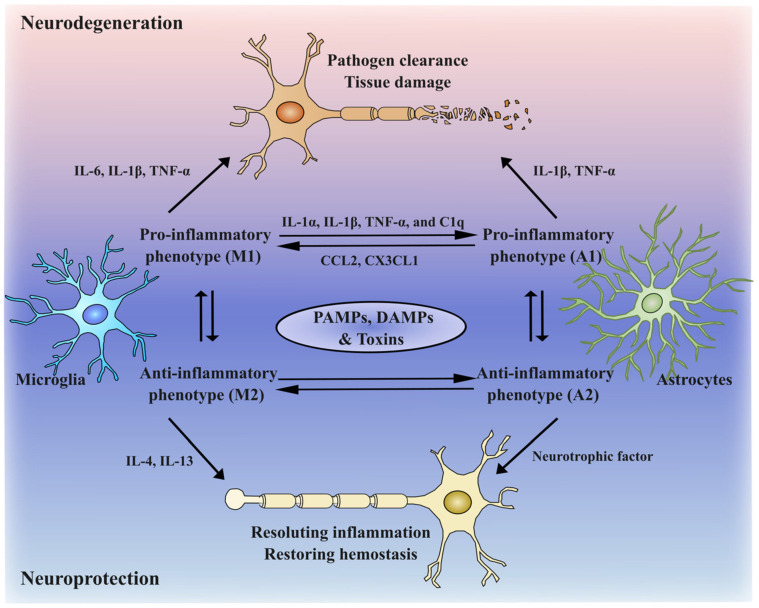
Schematic presentation of microglia and astrocyte polarization in neurodegenerative and neuroprotective environments. Upon stimulation by pathogen-associated molecular patterns (PAMPs), damage-associated molecular patterns (DAMPs, such as Aβ, α-syn, or tau), and toxins such as nitric oxide (NO), microglia are activated into the M1 proinflammatory phenotype. M1 microglia can interact with A1 astrocytes, which are also in a proinflammatory phenotype. Although microglia can transition from the M1 to M2 anti-inflammatory phenotype, in neurodegenerative diseases, such as Alzheimer’s and Parkinson’s disease, M1 microglia become more prevalent at injured sites in the later stages of the disease, while the immunoregulatory and repair functions of M2 microglia are suppressed.

Of particular significance to microglia is their ability to exhibit diverse phenotypes, such as compact round to hypertrophic with retracting branching in response to disease stages in the local microenvironment and a variety of activation pathways [46,47]. The simplistic M1 (proinflammatory effects) and M2 (anti-inflammatory effects) paradigms were initially introduced to classify microglial activation, similar to astrocyte classification (A1-A2). As depicted in Figure 1, M1 microglia secrete proinflammatory mediators, while M2 microglia remove apoptosis-related aggregates through phagocytosis [14,15].

Although this M1-M2 dichotomy has been criticized for oversimplifying the complex and heterogeneous nature of microglial activation, this model is still widely adopted for easy understanding. Emerging evidence from single-cell transcriptomic studies proved that microglia exist in a broad spectrum of dynamic states that are constantly interchanging within the in vivo environment [48,49,50]. Once microglia are activated in a primed state, they fail to return to a quiescent state, leading to sustained inflammation and potentially detrimental effects on neuronal survival [51]. Activation of TLR4 in microglia can induce necroptosis and exacerbate neuroinflammation [52]. Microglial necroptosis has been observed during the process of remyelination, which is associated with multiple sclerosis [53]. While microglia can transition from the M1 to M2 phenotypes, the underlying mechanisms involved in this process are complex and not yet fully elucidated [54]. The distribution of microglial phenotypes can vary depending on the NDD stage [55]. Moreover, proinflammatory microglia can interact with astrocytes exhibiting a similar phenotype through the secretion of cytokines, including IL-1α, IL-1β, TNF-α, and C1q [56]. These proinflammatory astrocytes can then activate microglia by secreting CCL2, CX3CL1, CXCL10, GM-CSF, and IL-1, perpetuating the inflammatory response in the CNS. The intricate nature of microglia may explain the lack of effective anti-inflammatory drugs to date.

### 2.4. Persistent Neuroinflammation

Chronic neuroinflammation has been observed in the brains of patients with NDDs [57]. It is characterized by a prolonged and often self-sustaining inflammatory response that persists long after the initial injury or insult. The ongoing nature of chronic inflammation is supported by the persistence of microglial activation, impaired clearance of damaged cells or debris, and the presence of persistent stimuli that continue to activate the immune response [58].

Emerging evidence has shown that the ubiquitin–proteasome system, autophagy and complement system are correlated with microglial priming [59]. This self-amplifying response gradually contributes to weak synapses, which is the hallmark of several NDDs [60]. This is especially evident in MS, and the excessive activation of microglia leads to the destruction of myelin [61].

Disease-associated microglia (DAM) are transcriptionally distinct microglial profiles that are specific to NDDs, including AD, PD and MS [62]. The gene expression changes in DAM have been investigated and revealed to vary depending on the specific NDDs being studied, as shown by transcriptome and genome-wide association studies [63]. For example, in Alzheimer’s disease, DAM have been characterized by the upregulation of genes such as apolipoprotein E (APOE) and triggering receptor expressed on myeloid cells 2 (TREM2) while downregulating genes involved in synaptic function and homeostasis [64,65]. In the case of MS, microglia associated with distinct lesion types have been found to express higher levels of genes involved in immune cell interactions, such as MHC class I and II molecules, compared to normal-appearing white matter [66]. High levels of MHC class II antigen expression have also been observed upon activation of α-syn in models of PD [67]. Hence, a better understanding of the alterations in gene expression patterns of DAM has provided valuable insights into the underlying pathogenesis of NDDs and the development of therapeutic interventions.

### 2.5. Therapeutic Applications of Immunomodulatory Medications in NDDs

Although conventional strategies for selectively targeting misfolded and aggregated proteins have been employed in treating NDDs, accumulating evidence indicates that chronic neuroinflammation also substantially contributes to their pathogenesis. In view of this, a summary of the commonly used classifications of neuroimmunomodulatory agents for the treatment of neurodegenerative diseases is provided in Table 1.

The use of nonsteroidal anti-inflammatory drugs (NSAIDs) (including ibuprofen, indomethacin, aspirin, meclofenac, and flurbiprofen) has been suggested by previous epidemiological studies as a potential therapeutic approach for conferring neuroprotective effects in the pathologies of NDDs such as AD and PD [68,69]. NSAIDs may exert direct and indirect effects on microglial activation through various mechanisms. These include the activation of peroxisome proliferator-activated receptor gamma (PPAR-γ) and inhibition of nuclear factor kappa B (NF-κB), activator protein 1 (AP-1), and mitogen-activated protein kinase (MAPK) p38 signalling pathways, leading to alterations in gene expression typically associated with microglial activation. Additionally, NSAIDs have been proposed to inhibit cyclooxygenase (COX) activity, promote amyloid-β clearance, and interfere with secretase activity in neurons, further modulating microglial function [70]. The use of NSAIDs as a preventive strategy for AD has been previously suggested. However, the hypothesis for this purpose has been questioned by updated findings from a meta-analysis of cohort studies and randomized control trials, which have not yet provided sufficient evidence to support the benefits of NSAIDs in reducing AD prevalence among patients [71,72]. The available results were inconsistent and conflicting, with no clear evidence demonstrating a direct beneficial effect of NSAIDs for preventive purposes [71,73]. Although one of the NSAIDs, mefenamic acid, has demonstrated efficacy in relieving menstrual pain and suppressing neuroinflammation and memory loss in experimental amyloid-β-induced AD in mice, the potential neurotoxic effects of NSAIDs, including aspirin, in AD patients may be a concern [74]. In the later stages of NDDs, NSAIDs may have detrimental effects, potentially as a result of their inhibitory activity on activated microglia.

The immunoregulatory drugs used in the treatment of neuroinflammation include a variety of agents, including monoclonal antibodies, biologic drugs, small molecule drugs, and steroid hormones such as glucocorticoids [75]. In fact, the most commonly used immunoregulatory drugs and those currently being investigated for their functions in neuroinflammation are monoclonal antibodies (including natalizumab, rituximab, and anti-CD20 monoclonal antibodies). Natalizumab is a monoclonal IgG4 antibody that was approved by the FDA in 2004 for the treatment of relapsing-remitting MS. Natalizumab can inhibit the transmigration of leukocytes throughout the BBB into the brain by blocking very late antigen (VLA)-4 interactions. This intervention mediates neutrophil invasion and interacts with microglia, which can ameliorate inflammatory lesions in MS patients [76]. However, mounting evidence suggests that the use of various monoclonal antibody immunotherapies, including but not limited to natalizumab, may be associated with an increased risk of progressive multifocal leukoencephalopathy (PML), a subacute cerebral inflammatory disorder primarily observed in immunocompromised patients [77].

As our understanding of neuroinflammation continues to expand, various novel strategies involving monoclonal antibodies have been developed, each with remarkable specificity and efficacy. Examples of such strategies include B-cell depletion, which has been demonstrated to drive the neuroinflammatory response in NDDs [78]. Notably, the use of anti-CD20 antibodies such as ofatumumab has achieved remarkable success in the treatment of MS [79].

Etanercept is a biological drug (fusion protein that contains an antibody Fc portion) that acts as a proinflammatory cytokine TNF-α inhibitor. A previous clinical trial investigated the use of etanercept via perispinal administration in patients with mild, moderate, and severe cases of AD. The trial reported sustained cognitive improvement for up to six months in these patients, indicating the potential therapeutic effects of etanercept in AD [80]. In 2015, a randomized clinical trial of 24 weeks of subcutaneous etanercept for AD patients showed no significant changes in cognitive, behavioural, or global function [81]. The conflicting results observed in studies investigating cytokine levels in AD may be attributed to variations in the disease stage and the potential significance of cerebrospinal fluid (CSF) cytokine studies. However, preclinical data suggest that TNF-α can initiate an inflammatory cascade that may have either neuroprotective or neurotoxic effects on disease progression, depending on its timing, location, or cellular source [82].

Due to their high accessibility, low toxicity, and potent anti-inflammatory properties, natural products (including alkaloids, steroids, terpenoids, flavonoids and polyphenols) have been proposed as potential agents for preventing and mitigating neuroinflammation [35,83,84,85]. These natural products share the common feature of possessing a multitargeted approach and exert their potential neuroprotective properties by either inhibiting translocation of NF-kB dimers into the nucleus, regulating the release of proinflammatory cytokines and the production of inflammasomes, or inducing anti-inflammatory effects [86]. A recent systematic review concluded that most polyphenols from plant sources can modulate microglial polarization via the TLR4/NF-κB pathway, exerting anti-inflammatory effects in ischaemic stroke and other neurological disorders [84]. For instance, resveratrol is a polyphenolic compound that is naturally present in grapes, berries, and other medicinal plant sources. In a pilot study of treatment after ischaemic stroke, administration of resveratrol was found to reduce infarct volumes and suppress levels of NO, IL-1β and TNF-α [87,88]. It also promotes M2 polarization in microglia by inhibiting the NF-κB pathway and activating peroxisome proliferator-activated receptor-γ coactivator (PGC-1α), and it has been found to preserve the integrity of the blood–brain barrier (BBB) [89,90]. Nonetheless, the low bioavailability of phytochemicals poses a significant challenge to their efficacy in the treatment of neuroinflammation. Previous pharmacological experiments have demonstrated that the oral bioavailability of resveratrol is typically less than 1% due to extensive metabolism in the intestine and liver [91]. The clinical use of curcumin has also been reported to be limited by its poor water solubility and large molecular size. Furthermore, the promiscuous nature of phytochemicals makes them highly susceptible to interactions with off-target molecules, which may lead to unintended effects.

Other than the above interventions, there are numerous alternative therapeutic approaches available for the regulation of neuroinflammation, including the use of antibiotics and other modalities. Minocycline is a second-generation tetracycline antibiotic known for its ability to inhibit the activation of microglia and astrocytes [92]. The administration of minocycline was found to exert neuroprotective effects by inducing polarization of microglia towards an M2 phenotype while inhibiting M1 polarization [93,94,95]. In a subsequent experiment, intraperitoneal administration of minocycline saline was found to improve spatial memory and promote adult neurogenesis in the rat hippocampus through STAT1/STAT6 pathways during a 48-h sleep deprivation model, which is known to be associated with neuroinflammation [96]. Moreover, minocycline has been reported to bind and disassemble preexisting fibrillogenic structures of amyloid, as demonstrated in studies involving synthetic peptides of human PrP and Aβ [97,98]. Doxycycline (tetracycline antibiotic) shares similar antiamyloidogenic properties, particularly in its ability to act against soluble β-amyloid oligomers. This property has been demonstrated to restore memory and reduce neuroinflammation in mouse models of AD [99]. The anti-neuroinflammatory effects of interventions in previous animal models of neurodegeneration are undeniable, yet there have been criticisms that their ability to reverse neuronal damage may be insignificant [86,100].

Overall, there is currently no effective way to treat neuroinflammation. Mixed results and adverse effects have raised concerns about the suitability of the interventions for treating neurological disorders. Moreover, these compounds are criticized for being administered systemically without spatial or temporal control [36]; in particular, phytochemicals may have the potential to interact with off-target molecules. Precise delivery of medication to the CNS is urgently required for effective management of complicated neuroinflammation.

**Table 1:** Summary of common classifications of neuroinflammation-modulating agents for NDDs.

Classification	Feature	Example	References
1. NSAIDs	Controversial results in clinical data Not recommended for preventative purposes Severe side effects (e.g., gastrointestinal toxicity, cardiovascular risk and renal impairment)	Aspirin, Celecoxib, Naproxen, Mefenamic acid	[70]
2. Immunomodulatory drugs			
a. Monoclonal Antibodies	High specificity and versatility Some of them approved by FDA Adverse effects such as PML were observed	Natalizumab, Rituximab, Ofatumumab	[76,79]
b. Biologic drugs (e.g., TNF-α inhibitor)	High variations among disease stages	Etanercept	[80]
3. Phytochemical compounds	• Emerging field with numerous advantages (e.g., multitargeted, potent anti-inflammatory effects, readily accessible and negligible toxicity) • Usually, low bioavailability • Limited clinical data available • Possibly interact with off-target molecules	Resveratrol, Curcumin, Quercetin	[84,101]
4. Others			
Tetracycline Antibiotics	Direct effects on inhibition of activated microglia and astrocytes Anti-amyloidogenic properties May be insufficient to reverse neuronal loss	Minocycline, Doxycycline	[86,99]

## 3. Aptamers

Aptamers are single-stranded nucleic acid DNA or RNA, ranging in length from 20 to 100 nucleotides (nt) and function like monoclonal antibodies [102,103]. The term “aptamer” is derived from the Latin word “aptus” (fitting) and the Greek word “meros” (region). This nomenclature reflects the extraordinary ability of aptamers to selectively bind and recognize specific targets by their unique secondary and tertiary conformations [104,105]. The binding affinity of aptamers is attributable to their diverse nucleotide sequences and their folding tendency to form diverse structures, including stem–loop, bulges, pseudoknot, G quadruplex and hairpin structures, which enables them to distinguish subtle target molecules [106,107,108]. Moreover, the dissociation constants of aptamers are typically within the picomolar to nanomolar range, indicating that they are capable of functioning effectively at low concentrations [109,110].

Given the similarities between aptamers and antibodies, these two classifications of drugs are often compared in the development of therapeutic agents [111]. Both can selectively bind to target molecules with remarkable affinity and specificity. The structural binding between an aptamer and its target resembles the antigen-antibody interaction and is mediated by noncovalent interactions, including van der Waals forces, hydrogen bonding, hydrophobic interactions, and ionic interactions [112,113,114]. Aptamers are generally considered to offer distinct advantages superior to monoclonal antibodies as therapeutic agents in terms of their numerous pharmacokinetic advantages, including thermostability, minimal immunogenicity, prolonged storage life, facilitated transportation and minimal batch-to-batch variation [115].

Small nucleic acid aptamers offer a highly versatile platform for recognizing a wide range of molecular targets, allowing them to adhere to the cell surface and even insert themselves into the crevices present on the surfaces of their targets. This property enables precise target recognition and identification of previously undiscovered target sites [116]. Currently, aptamers have been developed to target various molecules, including heavy metal ions, metabolites, chemical compounds, peptides, proteins, viruses, bacteria, whole cells, and even mammalian cells, demonstrating their broad potential applications in various fields [117,118,119,120].

### 3.1. SELEX

**Figure 2:**
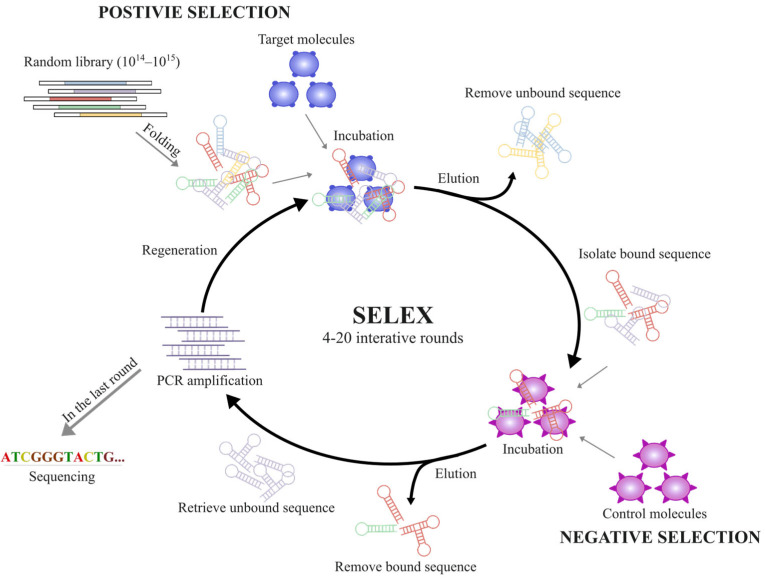
Schematic diagram of the SELEX process. The SELEX process involves several key steps: (1) incubation of oligos with target molecules, (2) repetitive elution for removing weak and nonspecific interactions, (3) partitioning and amplification of the bound aptamer sequences via polymerase chain reaction, and (4) regeneration of single-stranded oligonucleotides to be used in subsequent rounds. This process is terminated once there is no further increase in binding affinity, indicating that the aptamer-target interactions have reached their peak.

The in vitro generation of aptamers known as the Systematic Evolution of Ligands by Exponential Enrichment (SELEX) was first introduced by Tuerk and Gold in the early 1990s (Figure 2) [121,122]. With the aim of identifying high affinity aptamers, a variety of random sequences (1014–1015) are subjected to competitive binding with a target molecule and elution via multiple rounds [123]. The entire SELEX process typically involves 4–20 iterative rounds, with progressively increasing selection stringency until maximal affinity is achieved [124]. Negative selection involves interaction with competitors with a high degree of homogeneity or an empty support matrix to remove nonspecific and false-positive binding. The SELEX technique allows for the generation of aptamers without prior knowledge of the target molecules [125,126]. It is typically generated through Protein-SELEX or Cell-SELEX [127,128], in which specific cells or proteins of interest are targets. For example, S6-1b aptamers recognize the SHG44 glioma cell line and human astrocytes without prior knowledge, while the precise binding sites were elucidated later by mass spectrometry [129].

The SELEX technique has been augmented with innovative methods, such as magnetic bead-based competitive [130], fluorescence-activated cell sorting [131] and microfluidic chip-based selection [132], and has even been utilized in vivo. Current SELEX methods offer significantly rapid screening processes and identification of aptamers for previously unestablished targets, depending on their intended applications. This unique feature makes aptamers a valuable tool for addressing not only global infectious issues such as COVID-19 and Ebola but also other health crises, such as the lack of effective interventions in NDDs [119,133].

### 3.2. Modifications of Aptamers

**Figure 3:**
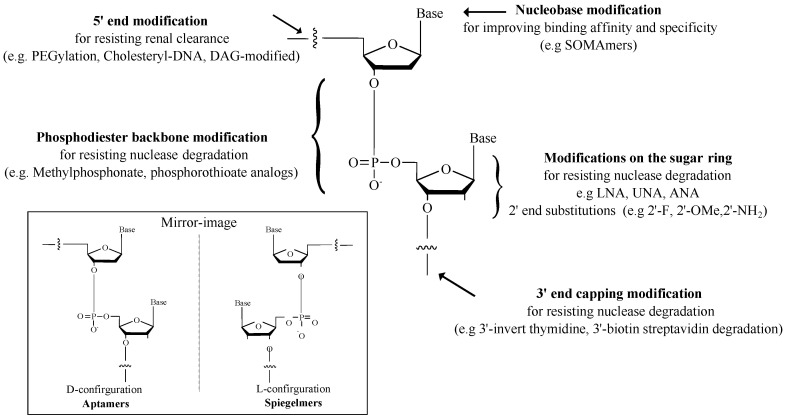
The strategies of chemical modifications employed on aptamers are classified based on the sites of modification, including the phosphodiester backbone, nucleobase, sugar ring, and 3′ and 5′ positions of the oligonucleotide, as well as the purpose of modification.

Following the SELEX process, the aptamer candidates have been primarily identified. However, natural oligonucleotides are inherently susceptible to nuclease attack and are rapidly excreted through renal filtration due to their small size (6–30 kDa) [134]. Pharmacokinetic studies have indicated that unmodified aptamers generally have short half-lives of 2–10 min both in vivo and in vitro in tissue culture [135], which greatly impacts their efficacy as drugs. These limitations are addressed through in-SELEX or post-SELEX modifications of the phosphodiester backbone, nucleobase, sugar ring, and 3′ and 5′ positions [105], as summarized in Figure 3. For example, 3′-terminal capping with inverted thymidine is commonly used to enhance stability and resistance in human serum [136,137]. Furthermore, the unique conformation of spiegelmers (L-deoxy-oligonucleotides as mirror images of D-form) provides an advantage in preventing high attacks from ribonucleases due to their absence in nature [138]. In addition to additional modifications, truncation of nonfunctional sites has been employed to improve the specificity and affinity of aptamers [139]. Together, aptamer modification has the potential to confer several benefits, including extending half-lives and enhancing stability and accessibility to target molecules [105,140].

**Figure 4:**
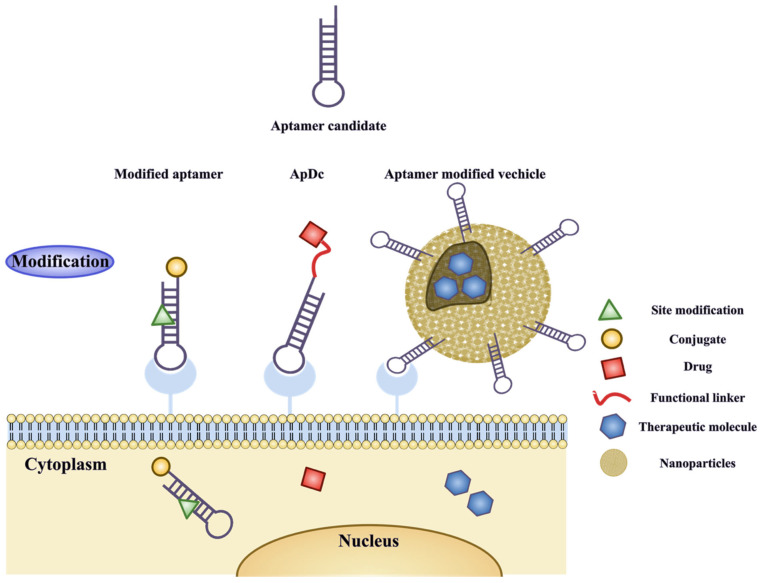
Aptamer-based therapeutics require modifications to achieve therapeutic effects. As previously mentioned, aptamer candidates are prone to degradation and excretion and are often chemically modified and conjugated with agents such as 2′-Fluoro, 2′O-methyl and 2-Thiouridine. Another ApDC approach is to conjugate aptamers with various therapeutic agents, such as chemotherapeutics, nucleic acids, proteins/peptides, photosensitizers, and photothermal agents. Functional linkers are designed to facilitate the formation of stable conjugates, enabling controlled drug release in specific tissues or cells. One emerging approach for aptamer delivery is to encapsulate them within nanoparticles such as liposomes, gold nanoparticles (AuNPs), and metallic nanoparticles, which can protect therapeutic agents from degradation and enable more specific targeting to particular cells.

Apart from modifications to the therapeutic effects of the aptamer per se, high-affinity aptamers can be engineered into aptamer-drug conjugates (ApDCs) and aptamer-nanoparticle drug vehicles to facilitate the targeted delivery of therapeutic agents to specific tissues or cells (as shown in Figure 4). This feature was initially utilized in cancer therapy due to its potential to reduce off-target side effects and enhance therapeutic efficacy against cancer cells. Subsequently, it has been developed for use as a diagnostic and therapeutic agent for various diseases beyond cancer [141,142].

Moreover, aptamers can bind to target molecules both extracellularly and intracellularly, and they are typically internalized into cells via mechanisms such as micropinocytosis and endocytosis. Clathrin-mediated endocytosis (CME) and caveolae-mediated endocytosis (CvME) are common mechanisms by which aptamers are internalized into most eukaryotic cells [143,144]. Of these pathways, the predominant mechanism is clathrin-dependent endocytosis [145], which involves the formation and detachment of clathrin-coated vesicles that transport aptamers into cells [146,147]. Partial aptamers that enter cells via clathrin-dependent endocytosis may become dysfunctional due to degradation by endosomes and lysosomes. For example, the R13 aptamer targeting ovarian cancer cells has been proven to be internalized via clathrin-dependent endocytosis [148].

### 3.3. Limitations of Aptamers

The discovery of aptamers is often impeded by the difficulty of generating aptamers with adequate binding capacity or specificity, as well as the subsequent modification, evaluation, and optimization processes [149]. Experimental conditions, including the temperature, buffer components (ions, ionic strength, and pH), and other factors, can significantly impact the structures of aptamers and their interactions with targets, leading to potential false positives [150].

The process of iterative rounds of selection, optimizing the modification, followed by experimental validation, is obviously labour intensive and costly. Moreover, aptamers may pose potential toxicities by themselves or with newly added chemical modifications, particularly with continuous administration of interventions [135]. For example, PEGylation, which is often used to enhance the bioavailability of aptamers, has been reported to cause severe immune responses in a phase III clinical study [151]. Last, the administration of aptamers is typically restricted to injection, which may limit their use in certain applications.

The adverse effects associated with therapeutic oligonucleotides are significant and warrant careful consideration, including coagulation inhibition, complement activation, immunostimulation and tissue accumulation [152,153]. The tissue accumulation phenomenon can result from the high affinity of oligonucleotides towards specific tissues, as well as the relatively large size of conjugation modification, which can hinder their renal clearance and hepatic metabolism [154]. However, these effects are generally considered nonadverse due to their reversible nature upon cessation of treatment [153].

Compared to other therapeutic agents, there is limited information available on the pharmacokinetic properties, including absorption, distribution, metabolism, excretion, and toxicity (ADMET), of aptamers, particularly in disease stages [155]. Animal models used in aptamer-based therapy are often limited to mice, which may not accurately recapitulate human disease conditions due to differences in genetics and regenerative ability [156]. For instance, the unexpected results of the NOX-A12 aptamer were due to the low levels of CXCL12 secretion by human bone marrow stromal cells (BMSCs), whereas murine BMSCs secrete higher levels of CXCL12, resulting in treatment disparities [157].

Despite their limitations, aptamers remain a desirable platform for a variety of therapeutic biomedical applications due to their easy engineering and highly selective features [141]. Until now, the application of aptamers in neuroscience has been advanced in diagnosis, detection, imaging, neurotransmitter visualization, and therapeutics in brain-related diseases [104]. In the following sections, we will discuss the developed aptamers targeted to brain delivery, their function acting on neuroinflammation in the context of NDDs and finally their opportunities and challenges in detail.

## 4. Aptamer-Based Targeted Brain Delivery

The therapeutic effects of conventional medications, such as small molecule drugs, larger molecules, therapeutic peptides, or inhibitors, are often unsatisfactory in treating neurological disorders due to challenges in transporting them across the BBB [158]. As previously discussed, the systemic mechanism of action of those compounds, without precise spatial or temporal control, has raised significant concern about off-target effects. For instance, glucocorticoids are widely used in the treatment of autoimmune disorders, including MS, due to their potent anti-inflammatory and immunosuppressive properties [159,160]. Unfortunately, these drugs have side effects on multiple organ systems, such as musculoskeletal and gastrointestinal organs [161,162]. These circumstances underscore the need to explore alternative delivery methods that can target precisely and exert effective therapeutic interventions. The utilization of aptamers may present a promising solution for drug delivery to the brain and as a therapeutic intervention in NDDs.

### 4.1. Route of Administration

The administration route is a pivotal factor that has a consequential impact on the ADMET properties of drugs, which in turn may affect their tissue accumulation and therapeutic effects [163]. The approved aptamers are generally administered through intravitreal, intrathecal, local, subcutaneous, intramuscular, and intravenous routes [164]. The development of aptamer-based therapeutics should prioritize the preservation of the integrity of various nucleic acids in complex living environments, particularly in the context of targeted delivery [164]. The administration routes for drug delivery into the brain have raised concerns in the field of neuropharmacology, whether invasive (intrathecal, intracerebroventricular injection, convection-enhanced delivery and intracranial implantation) or noninvasive approaches (intranasal, subretinal, intravenous).

From the perspectives presented in systematic reviews, each method has its own advantages and disadvantages [36,149,153,165]. Conclusively, noninvasive administration appears to be a preferred approach over invasive methods for the treatment of brain pathologies. The challenges are particularly evident in the case of NDDs that are located deep within the brain and the potential risks of infection associated with invasive methods [166,167]. Additionally, the limited bulk flow of cerebrospinal fluid (CSF) can restrict the efficiency of therapeutic delivery to the brain via invasive methods [168].

Over the past few decades, there has been substantial progress in the field of aptamer-based delivery strategies for targeting the brain. In this section, we will discuss these advancements and their implications in the treatment of neuroinflammation-related disorders.

### 4.2. Can Aptamers Penetrate the Blood–Brain Barrier?

Following injection, aptamers must traverse multiple biological barriers to reach their intended pharmacological targets [153]. The blood–brain barrier (BBB) is the most significant obstacle among these barriers to delivering therapeutic aptamers to the brain [169].

The blood–brain barrier is a semipermeable membrane that separates the circulating bloodstream from the extracellular fluid in the CNS. It can serve as a crucial guard that protects the brain from harmful substance threats such as toxins in blood, filters harmful substances from the brain into the bloodstream and maintains internal homeostasis [149,170,171]. This highly selective structure is composed of capillary endothelial cells with tight junctions and specialized transport proteins embedded on the luminal side, as well as astrocytic end feet surrounding the capillaries to support them [172,173].

The efficacy of aptamer-mediated delivery across the BBB is dependent on several factors, including the specific aptamer sequence, its molecular weight, and the presence of competing ligands [149]. The BBB allows for transcytosis of high lipid solubility (with a logP value of approximately 2.1) and small molecules (<400–500 Da) [174]. Considering the physicochemical properties of unmodified aptamers, such as their negatively charged and lipophobic nature, in addition to their relatively large molecular weight (100 bp ssDNA for approximately 30 kDa), most aptamers cannot traverse the BBB and reach the brain effectively [153,175]. Therefore, measurements such as truncation and conjugation with lipophilic agents can enhance the drug delivery of aptamers across the BBB.

Apart from truncation and lipophilic conjugation measurements, researchers have demonstrated ingenuity in developing aptamers that bypass the BBB through transport by exosomes or encapsulation within nanoparticle carriers, such as nanoliposomes, and selectively targeted binding to membrane proteins [153,176].

### 4.3. Aptamers Encapsulated by Exosomes to Bypass the Blood–Brain Barrier

The current approach for delivering aptamers to the brain involves encapsulating them in exosomes to bypass the blood–brain barrier (BBB). Exosomes, which are extracellular vesicles released by cells, are increasingly being utilized as a mechanism for drug delivery [177]. These vesicles can be loaded with various molecules expressed by cultured cells and then isolated from the cell culture medium. By exploiting the natural intercellular trafficking properties of exosomes, these vesicles can be used to deliver a wide range of cargo molecules, including nucleic acids, proteins, and lipids, to target cells [178].

The F5R1 DNA aptamer was developed in 2018 and its effects on preventing the aggregation of α-synuclein and promoting its degradation for the treatment of PD were demonstrated [179]. However, a subsequent study of the F5R1 aptamer revealed its preferential binding to fibrillar α-synuclein, rather than to the monomeric form. To enhance the therapeutic efficacy of the F5R1 aptamer, it was encapsulated in exosomes that were isolated from the culture medium of HEK293 cells. These exosomes were specifically modified to express the neuron-specific rabies virus glycoprotein (RVG) on their surface, enabling targeted delivery to neuronal cells. As a result, RVG-decorated exosomes containing the F5R1 aptamer were observed to undergo retrograde transport and transsynaptic transmission into the CNS via the axons and synapses of peripheral neurons following intraperitoneal administration [180].

### 4.4. Aptamers Targeting the Membrane Transferrin Receptor (TfR)

The transferrin receptor (TfR) targeting approach has emerged as a highly active domain of drug delivery to the CNS in recent years. In a pioneering experiment in 2013, Cheng and his colleagues conducted an in vivo selection of therapeutic aptamers for brain penetration. An RNA library was injected into mice via the tail vein, followed by brain collection, ribonucleic acid extraction, amplification, and selection. The selected aptamer, A15, was found to bind initially to mouse endothelial cells and successfully penetrate the blood–brain barrier [181].

The TfR is expressed on the endothelial cells of the BBB in healthy individuals and on tumour cells and is commonly used as a drug trafficking target for aptamers [182,183,184]. The transferrin transport pathway has been exploited in multiple rodent studies to carry therapeutic payloads into the brain [153].

One of the representative examples of aptamer-modified vesicles is TfR aptamer-functionalized liposomes (Apt-LP) developed by Zhang et al. in 2021. Apt-LP is a functional aptamer that specifically binds to TfR, while the therapeutic payload (AchE reactivator obidoxime) is encapsulated with a liposome to facilitate delivery to the brain against paraoxon (POX) poisoning. Once this interaction occurs, the liposome is rapidly internalized into endothelial cells via receptor-mediated endocytosis, allowing for intracellular release of the therapeutic payload. In comparison to without-aptamer-targeting liposomes and a scrambled sequence-modified liposome, the uptake of Apt-LP by brain endothelial cells was significantly 40% higher. Liposome encapsulation also contributes to drug delivery into the brain. This result was confirmed by in vitro penetration BBB transwell assays and in vivo assays such as biodistribution studies and ex vivo fluorescence images [185]. On the other hand, the application of bifunctional aptamers in ApDCs represents a promising strategy for targeted drug delivery to the brain. Unlike traditional drugs, two aptamers are conjugated with a flexible linker (as previously mentioned in Figure 4) that enables simultaneous binding to two or more specific targets with high affinity.

**Figure 5:**
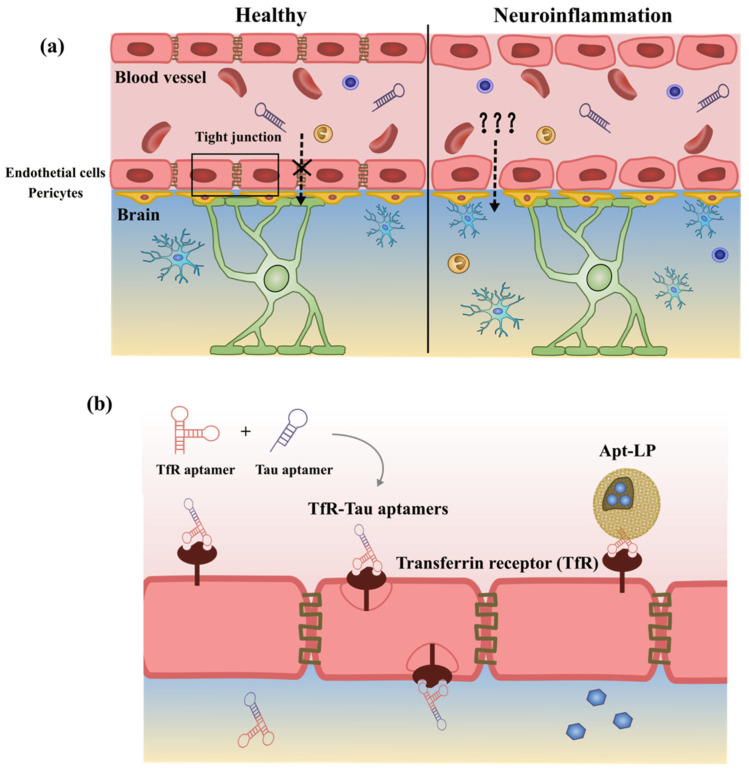
(**a**) The transport of therapeutic aptamers across the blood–brain barrier (BBB) in healthy and neurodegenerative brains. In a healthy individual, most therapeutic aptamers are restricted from entering the brain due to the tight junctions of endothelial cells unless the specific requirements have been fulfilled. On the other hand, the transport of therapeutic aptamers across the BBB in the context of neuroinflammation and neurodegenerative diseases (NDDs), where the BBB is impaired, poses uncertainty to drug distribution and may contribute to the accumulation of aptamers in the brain. (**b**) The enlarged schematic representation of aptamer-mediated drug delivery strategies for entering the BBB, including ApDc and aptamer-modified vehicles. The transferrin receptor (TfR) is expressed on the luminal surface of specific vascular endothelial cells, which are the target port for transport agents into the brain. Current approaches include dual aptamers targeting multiple receptors (such as the conjugation of TfR and Tau aptamers) and the application of liposome-conjugated TfR aptamers (Apt-LP) that have been shown to successfully pass through the BBB.

In 2020, Li and her colleagues developed a novel dual aptamer system (TfR-Tau aptamer) comprising a TfR aptamer and a circular Tau aptamer. The TfR aptamer selectively targets the transferrin receptor on the BBB, while the tau aptamer binds to the tau protein and disrupts the tauopathy process in the brain (Figure 5). The TfR-Tau aptamer system demonstrated enhanced transcytosis capacity across the BBB compared to the TfR aptamer and Tau aptamer alone, as confirmed by rigorous experimental assays, including in vitro bEnd.3 transwell migration assays and ex vivo imaging techniques such as Cy5.5-fluorescence signals in organs at different time points (0.25, 1, 2, 4, 8 h) and confocal microscopy of dissected brain tissue [186]. More importantly, the TfR-Tau aptamer has progressed to animal experiments in traumatic brain injury (TBI). In these trials, mice were either administered circular aptamers at a dose of 200 nmol/kg once daily for five days, followed by evaluation of TBI-related protein levels in brain and serum samples, or received five weeks of circular aptamer treatment and subsequently underwent Y-maze testing with 2-min and 1-h intertrial intervals. The results of these experiments demonstrated a significant reduction in TBI-related protein levels in the brain, and the FPI model mice exhibited an increased time spent in the arms of the Y-maze after a 1-h intertrial interval [186]. A similar approach of dual aptamers has been employed in the treatment of brain cancer metastases. For example, Macdonald et al. developed a dual aptamer system in 2016 that targets cancer cells expressing epithelial cell adhesion molecules (EpCAM) and the transferrin receptor (TfR) [187].

These examples highlight the potential of aptamer-based drug delivery systems for the treatment of neurological disorders. ApDc systems offer a precise means of delivering therapeutic agents (including aptamers and others) to the brain, thereby increasing efficacy and minimizing off-target effects.

## 5. Diagnostic & Therapeutic Aptamers for Inflammatory Biomolecules in NDDs

### 5.1. Aptamers for the Detection of Neuroinflammatory Biomarkers

Neuronal biomarkers are particularly useful in detecting and monitoring the progression of NDDs, including metabolites, misfolded proteins (Aβ, tau protein, α-Syn) and neuroinflammatory mediators (IL-6, PDGF) in the brain [188]. Measuring biomarkers in biofluids such as blood and saliva can be useful in detecting various diseases in humans. However, biomarker characterization for NDDs is challenging because of the physical protection of the skull and the presence of the BBB, which restricts the passage of many molecules. Certain small peptides, such as protein fragments, are more likely to traverse the BBB and be detectable in circulation, while metabolites also represent an important group of biomarkers for neurological disorders [104]. Recent investigations have highlighted the versatility of aptamers in detecting multiple targets relevant to neuroinflammatory disorders in blood and CSF, demonstrating their potential as a valuable detection tool for the diagnosis and monitoring of such conditions [189].

SOMAscan is a biomarker discovery and clinical diagnostic platform for proteomic detection based on high-sensitivity aptamers and quantified by microarray technology developed by SomaLogic, Inc. (Boulder, CO, USA, https://somalogic.com/neurology/, accessed on 15 March 2023). It allows the detection of over 7000 different human protein analytes in biological matrices [190,191]. This technique has been applied for identifying potential diagnostic or prognostic biomarkers for AD and MS [192]. For instance, Timsina and her colleagues recently conducted comparative analyses on SOMAscan and immunoassay-based protein measurements for five pathological proteins (NfL, Neurogranin, sTREM2, VILIP-1, and SNAP-25) associated with neurodegeneration. SOMAscan showed comparable predicted performance for all biomarkers to traditional immunoassays, except for SNAP-25 and sTREM2 analytes [193].

An increasing number of studies have also explored the application of aptasensors for various inflammatory cytokines and biomarkers linked to neuroinflammation by measuring changes in physical or electrochemical properties, respectively [194,195]. An aptamer-based colorimetric assay was developed for the detection of interleukin-6 (IL-6) in a mixed mouse protein solution using gold nanoparticles coated with two complementary aptamers specifically bound to IL-6 [196,197]. To directly detect abnormal proteins in NDDs, highly selective Aβ-40 aptamer sequences were immobilized onto silicon electrode surfaces of fabricated sensors to detect concentrations of Aβ-40 peptide. Surprisingly, this study demonstrated highly sensitive and reliable detection of Aβ-40 peptides at concentrations as low as 0.1 pg/mL [2]. For the detection of Aβ aggregates, a highly sensitive dual-aptamer-assisted assay on a quartz crystal microbalance was used as a mass-sensitive sensing platform, which provided a simple and effective method for detecting Aβ40 aggregates [198]. Furthermore, the enzyme-linked aptamer photoelectrochemical biosensor can sensitively detect Tau-381 protein from a concentration range of 0.5 fM to 1.0 nM [195].

Altogether, the development of real-time electrical biosensors based on aptamer ligands offers high sensitivity and cost-efficiency for the diagnosis of NDDs.

### 5.2. Therapeutic Aptamers Targeting Hallmark Proteins in NDDs

The accumulation of misfolded proteins or peptide fragments in the brain and/or spinal cord is a common characteristic among NDDs [199]. Moreover, these misfolded proteins and other damage-associated molecular patterns (DAMPs) can initiate and sustain microglial activation, which may exacerbate the progression of the disease. Hence, aptamers targeting abnormal proteins may offer a sensible approach for ameliorating neuroinflammation, akin to extinguishing the “flame” of neuroinflammation at its outset in NDDs. Here, we will discuss prominent misfolded proteins and their corresponding therapeutic aptamers.

#### 5.2.1. Aptamers against Aβ and BACE1 in AD

The deposition of amyloid-beta (Aβ) plaques in the brain is a hallmark of Alzheimer’s disease (AD) and is considered a toxic early event in disease pathogenesis [200]. Aβ is a 36–43 amino acid peptide fragment derived from amyloid precursor protein (APP) through sequential cleavage by γ- and β-secretases, such as beta-site amyloid precursor protein cleaving enzyme 1 (BACE1) and BACE2. In comparison to Aβ40, Aβ42 has been observed to display greater neurotoxicity and a faster aggregation rate, thus suggesting that it is a primary therapeutic target. Excessive production or impaired clearance of β-amyloid peptides can result in the development of amyloid plaques, also known as senile plaques. These plaques can initiate a cascade of deleterious events, including neuroinflammation, oxidative stress, and synaptic dysfunction, ultimately resulting in neurodegenerative disorders in affected individuals.

To our knowledge, there have been over ten reported anti-Aβ aptamers since as early as 2002, and most of them target the less neurotoxic Aβ40, such as β55 (RNA aptamer), KM33 (RNA aptamer), E2 (RNA aptamer) and RNV95 (DNA aptamer) [118]. Murakami and his coworkers successfully generated aptamers against Aβ oligomers in 2020. Specifically, they developed RNA aptamers named E22P-AbD4, -AbD31, and -AbD43 that bind to Aβ42 protofibrils. Histological studies were performed to evaluate the efficacy of these aptamers in two mouse models of AD, Tg2576/PS2 and AppNL-G-F/NL-G-F, and all three aptamers stained diffuse oligomeric aggregates. These results suggest that the oligomeric aggregates of Aβ formed in the brains of both mouse models shared similar protofibril-derived aptatopes [201,202].

As previously mentioned, amyloid-β (Aβ) is generated through the sequential cleavage of APP by γ- and β-secretases, with the latter enzyme (BACE1) emerging as a promising therapeutic target for AD. A previously identified DNA aptamer against BACE1 exhibited high affinity and specificity and significantly inhibited BACE1 activity in an AD cell model [203]. In a subsequent study, they investigated the inhibition of amyloidogenic pathway effects of the A1 aptamer in a mouse model. Four-month-old Tg6799 mice were administered intracerebroventricular injections of A1 aptamer or a scramble aptamer. Strikingly, behavioural experiments revealed that mice treated with aptamer A1 exhibited improved cognitive performance. Western blot analysis revealed a significant reduction in BACE1 and soluble amyloid precursor protein β (sAPPβ) expression in A1-treated mice. Moreover, aptamer A1 significantly decreased Aβ42 content, as well as the number and density of senile plaques in AD mice [204].

#### 5.2.2. Aptamers against α-Syn in PD

α-synuclein oligomers are recognized as significant contributors to the pathology and onset of PD and other synucleinopathies and are closely associated with the presence of Lewy bodies, which are a hallmark feature of these diseases [199]. Patients who suffer from PD frequently manifest involuntary movements, impaired balance and coordination, and may ultimately experience impaired speech. Oxidative stress is a significant contributor to the degeneration of the nigrostriatal pathway and is implicated in the formation of α-Syn aggregates. Interestingly, α-syn exhibited unexpected cross-reactivity with Aβ40 oligomers, which is a different amyloidogenic protein associated with AD.

DNA aptamers that target soluble oligomeric α-synuclein, termed T-SO517, were developed by Ikebukuro and colleagues in 2012. The researchers performed a competitive screening method to successfully identify eight aptamer candidates with high selectivity for α-synuclein oligomers among its monomeric, oligomeric, and fibrillar forms. Remarkably, these aptamers bind not only to α-syn oligomers but also to amyloid β oligomers, possibly because α-syn shares a common structural motif with amyloid oligomers [205]. Subsequently, two DNA aptamers, F5R1 and F5R2, were identified with high binding affinity and specificity to α-synuclein, with their potency as low as nanomolar dissociation values. Both aptamers were shown to effectively reduce α-synuclein aggregation in vitro and in cells and promote intracellular degradation of α-syn via the lysosomal pathway. As a result, the mitochondrial dysfunction and cellular defects resulting from α-synuclein overexpression were rescued. This represents a particularly promising finding, as it is the first study to demonstrate the efficacy of aptamers in inhibiting the aberrant cellular effects resulting from α-syn overexpression in cells [179].

## 6. Therapeutic Aptamers Targeting Neuroinflammation

There has been gradually increasing awareness of the potential role of aptamers in modulating the neuroinflammatory response in NDDs [206,207,208]. Uncontrolled and persistent neuroinflammation constitutes a pivotal factor in the progression of NDDs. Despite the predominant utilization of therapeutic measures targeting the aberrant protein processing pathway in neuroscience [118], investigational efforts concerning the therapeutic potential of aptamers in regulating innate immunity in neurological diseases, particularly NDDs, remain inconclusive.

Multiple aptamer-based interventions targeting inflammation, including inflammatory mediators or their receptors, have been explored in recent decades. Aptamers can act as antagonists that bind to specific receptors and block the biological response of the receptor to its pro- or anti-inflammatory mediators, thereby suppressing or counteracting their effects [209]. Most aptamers have been developed to specifically target a range of inflammatory mediators, including interleukins (IL-2, IL-6, IL-10, IL-11, IL-17, IL-23, IL-32), transforming growth factor-β (TGF-β) [210], tumour necrosis factor-α (TNF-α) [211], proinflammatory cytokines, interferon-γ (IFN-γ), chemokines (CCL2, IP-10), and their related receptors, as well as certain inflammatory autoantigens [212,213,214,215].

### 6.1. Aptamers Targeting Activated Microglia & Damaged Neurons

In recent years, aptamers have advanced to target a wide range of molecular targets, including activated microglia and damaged neurons. For example, ZH-1c aptamers have been selected to target microglia and further identified to bind with CD64, a transmembrane protein that is upregulated in polarized microglia in response to inflammatory stimuli, including LPS and IFN-γ [120]. Thus, the ZH-1c aptamer has the potential to target activated microglia in neuroinflammation.

In another previous study, PEG-M52 aptamers were selectively identified for their binding to CD200R, an immunoregulatory receptor expressed on microglia and other immune cells. The CD200-CD200R and CX3CL1-CX3CR1 signalling pathways are known to facilitate communication between neurons and microglia, which is believed to contribute to the homeostasis of microglia [216]. PEG-M52 aptamers were found to reduce microglial activation and inflammation by disrupting CD200-CD200R signalling [217]. Subsequently, the CD200R1 aptamer CCS13 demonstrated the strongest agonistic activity towards CD200R1, resulting in effective suppression of the induction of cytotoxic T-lymphocytes (CTLs) in in vivo immunosuppression [218]. Other costimulatory receptors, such as CD137 (4-1BB), were found to be expressed on neurons and astrocytes, which also developed corresponding aptamers [219,220].

### 6.2. Aptamers Targeting Proinflammatory Cytokines and Chemokines

Cytokines and chemokines are essential elements in mediating cell interactions and recruiting leukocytes, which can cause prolonged self-sustaining inflammatory responses in neuroinflammation [82,221]. Of the various cytokines implicated in the progression of dementia, emerging evidence suggests that IL-23 plays an important role in the pathogenesis of MS and age-associated inflammation in dementia [222]. IL-23, along with its p40 subunit, is closely related to microglial activation and amyloid β plaque formation [223]. Recent animal studies have shown that targeting IL-23 with anti-p40 antibodies can reduce amyloid β plaque formation and even restore cognitive performance in the APP/PS1 mouse model [224].

The IL-23 aptamer has been shown to have a neuroinflammatory suppressive effect by binding to macrophage stimulating 1 (MST1) kinase and blocking IL-23 in a mouse model of parathion-induced brain inflammation. Previous studies have demonstrated that MST1 kinase plays a role in mediating microglial activation in a stroke-induced model [225]. IL-23 aptamer treatment resulted in a significant reduction in the number of inflammatory infiltration foci, as evidenced by histological and immunohistochemical analyses. Furthermore, the IL-23 aptamer was found to reduce the absolute and relative numbers of MST1+CD4+ Th1 cells, as well as IL-23-producing cells, indicating a reduced neuroinflammatory response and protection of the brain from damage [215].

### 6.3. Aptamers Targeting Cell Surface Receptors

The presence of pathogens or other proinflammatory stimuli upregulates the expression of TLRs [226]. TLRs have been identified as major targets for neuroinflammatory disorders due to their wide expression on neuronal cells and their high correlation with all NDDs [227]. Notably, microglia expressing various TLRs are distributed across different regions of the brain, with a high preference for areas contiguous to the blood circulation, such as the meninges and circumventricular organs [228]. This phenomenon explains the rapid response of microglia to circulating endotoxin or lipopolysaccharide (LPS), as well as other ligands that activate TLRs [229].

Several aptamers targeting Toll-like receptors (TLRs) have been generated. As early as 2009, scientists developed an aptamer for Toll-like receptor 2 (TLR2), known as AP177, which has been demonstrated to significantly inhibit NF-kB activity and suppress cytokine secretion by up to 80% [230]. The R10-60 aptamer was subsequently developed as a TLR9 antagonist in 2017 [231]. The recognition of LPS patterns by TLR4 plays a critical role in the development of neurological disease [232]. TLR4 is highly expressed in microglia and sparsely expressed in astrocytes and neurons in the CNS [233,234]. Furthermore, the relationship between TLR4 and Ang II type 1 receptor has been confirmed to mediate BBB integrity, neuroinflammation, and autonomic dysfunction in spontaneously hypertensive rats [235]. Most recently, ApTOLL, a DNA aptamer antagonist to TLR4, has advanced to phase I clinical trials in humans (NCT04742062) for the treatment of myocardial infarction. Excitingly, no significant adverse effects or biochemical changes were observed in healthy volunteers across all dose groups and tested delivery patterns [236]. The use of ApTOLL has also been further expanded to acute ischaemic stroke in a phase Ib/IIa clinical trial (NCT04734548), where it has demonstrated neuroprotective effects for ischaemic stroke patients. Recent findings indicate that ApTOLL not only helps preserve and restore myelin and oligodendrocytes in demyelinated lesions, but also has therapeutic effects on the clinical symptomatology in an animal model of multiple sclerosis [237].

### 6.4. Aptamers Targeting the Complement System & Membrane Components

The component system, including C1, C3a, C3b and C5a, is related to microglial activation and neuronal damage in the progression of AD, MS and PD [238]. For instance, component 5a (C5a) is related to oxidative stress and inflammatory responses. The conjugation of anti-C5a aptamers (aC5a) with framework nucleic acids has shown promise in selectively reducing C5a-mediated neurotoxicity and effectively ameliorating oxidative stress in cerebral ischaemia–reperfusion injury [239].

PS is a membrane component that plays a role in the inflammatory process in neurons. Dysregulation of PS has been identified in numerous neurodegenerative and psychiatric diseases. Interestingly, supplementation with PS has been demonstrated to ameliorate age-related cognitive impairment in patients with Alzheimer’s disease (AD) and Parkinson’s disease (PD) [240]. In 2022, Su and colleagues developed PS-targeted aptamer-engineered exosomes loaded on biomimetic periosteum, which demonstrated significant angiogenesis in damaged nerves both in vivo and in vitro through the JNK3 MAPK pathway [241].

## 7. Emerging Opportunities and Complex Challenges of Aptamers in NDDs

### 7.1. Emerging Opportunities

To date, most aptamers are still in development or preclinical stages, being researched by academic and industrial scientists, with few exceptions on the market having received approval from the authorities [156]. The U.S. Food and Drug Administration (FDA) approved the first RNA aptamer (marketed as Macugen, Pfizer) for the treatment of age-related macular degeneration in 2004 [242]. Another successful example is the AS1411 aptamer targeting nucleolin, which is currently in phase II clinical trials for treating acute myeloid leukaemia (AML) and metastatic renal cell carcinoma (MRCC) [243,244,245]. These marked a significant milestone in the development of aptamer-based therapeutics, as the first mature aptamer product was approved for therapeutic use and a growing number of aptamers have entered clinical trials.

Despite their proven efficacy in treating immune-mediated inflammatory diseases, particularly those mediated by TLR signalling [246], immunomodulatory aptamers in the treatment of NDDs remain largely unexplored. This presents a unique opportunity for researchers to investigate their potential in a wider range of therapeutic applications. At present, the application of neuroinflammation in the context of preclinical research is limited, as further investigations are necessary to fully elucidate its potential applications and optimize its use. The fact that unintended immunogenic reactions in the brain environment can negatively impact healthy CNS tissues underscores the importance of using nonimmunogenic approaches to minimize such risks, making aptamers a promising option for the development of CNS therapeutics due to their negligible immunogenicity [247].

While B-cell or T-cell immune responses can be promising targets for intervention in several diseases, prolonged immunotherapy carries the risk of broad immune suppression or depletion. Ongoing research suggests that T-cell responses are regulated through cytokine-mediated up- or downregulation and inhibitory immune checkpoints [248]. These may offer a more targeted and effective approach. Recently, programmed cell death protein (PD-1) and its ligands have been shown to have suppressive effects on IFN-γ production, which can affect the progression of pathology associated with AD and improve brain and cognitive performance. Aptamers have been demonstrated as a successful example of targeting these molecules, such as aptPD-1 (DNA aptamers antagonizing PD-L1), in cancer immunotherapy [249]. However, the translation of validated aptamers to applications in NDDs remains a major concern and requires further investigation, as the effectiveness of these aptamers in treating NDDs may differ from their use in other contexts.

The utilization of aptamer-based targeted delivery systems has greatly expanded the potential applications of treatment in NDDs. On the other hand, a growing number of aptamers have been developed for regulating inflammation; for instance, the NOX-E36 aptamer is the only aptamer to have entered Phase I clinical trials for the treatment of chronic hepatic injury by liver macrophage infiltration and steatohepatitis [250]. High affinity aptamers can be incorporated into therapeutic agents in ApDCs and modified aptamer vehicles. This approach can be likened to a versatile skeleton key that has the ability to unlock multiple therapeutic doors, providing a valuable strategy for the development of novel therapeutic approaches. For example, dual TfR-Tau aptamers facilitate enhanced transcytosis capacity across the BBB, while Tau aptamers disrupt the tauopathy process in the brain.

The conjugation of aptamers with therapeutic agents has shown potential in improving the pharmacokinetic profiles of therapeutic agents. For example, Aptamin 320, a DNA aptamer conjugated with antioxidant vitamin C, protects vitamin C from oxidation when exposed to environmental stressors such as air, pH changes, high temperatures, and UV light [251]. Recent studies have demonstrated the potential of oral administration of aptamer conjugates in improving cognitive function and reducing oxidative stress in animal models. In a study using NXP032 (200 mg/kg), an aptamer conjugated with vitamin C, researchers found that it improved cognitive impairment and attenuated ageing-induced oxidative stress in 17-month-old female C57/BL6 mice by activating the Nrf2 signalling pathway [252]. Similarly, oral administration of NXP031 derivatives (200/4 mg/kg) was found to protect against dopaminergic neuronal loss and oxidative damage in a Parkinson’s disease mouse model after 8 weeks of treatment [190]. This approach is particularly promising, as vitamin C alone is highly susceptible to oxidation, which can compromise its therapeutic efficacy and limit its potential applications [251,252,253].

### 7.2. Complex Challenges

Some experts have suggested that current immunosuppressive and immunotherapeutic medications have failed to provide substantial benefits in NDDs due to potential targeting errors in treatment [16]. Most therapeutic aptamers were impulsively selected to be proinflammatory cytokines in early stages, while multifunctional cytokines could have neuroprotective or neurodegenerative effects within their microenvironment depending on the intensity and stage of disease [18,82,254]. For example, IL-1 is involved in mediating the effects of microglial activation on neuroplasticity. Excessive and prolonged IL-1 signalling can lead to neuronal damage and collateral damage. Despite the challenges associated with modulating cytokine activity, several aptamers targeting central cytokines have demonstrated success; for instance, the GS24-NFκB complex (combination of TfR aptamer and DNA decoy, which inhibits NFκB) diminishes inflammatory responses induced by TNF-α and exerts anti-inflammatory effects in the cerebral vasculature in an LPS-induced mouse model [255].

Cellular heterogeneity poses significant challenges due to the complex and dynamic nature of immune responses [207,256]. The use of aptamers targeting biomarkers on immune cells such as clusters of differentiation (CD) and receptors may appear to offer increased specificity and targeted effects. Notably, inflammaging is a dynamic process that continues to evolve over time, even during disease progression, and phenotypic and molecular changes have been observed in senescent immune cells [257]. Immune senescence is characterized by a progressive decline in immune competence and increased expression of inhibitory immune checkpoints and senescence markers [258]. For example, senescent markers of nonresting microglia states have been proposed, including high expression of CCL2 and VEGF [259]. Certain costimulatory receptors, such as CD28 and TCR signalling, may become impaired during T-cell senescence.

Given the complicated background of neurodegeneration, questions remain regarding the feasibility of treatment with immunomodulatory aptamers. While immune-modulating aptamers have shown promise in protecting experimental mice from acute neuroinflammatory models such as stroke by regulating body-brain signalling pathways, their effectiveness has not been well studied for prolonged usage. The use of immune-modulating aptamers for prolonged periods in vivo is also relatively rare. Importantly, neurodegeneration is a sustained inflammatory process, which presents a challenge for the development of effective and safe aptamer-based therapies [260].

Additionally, the pathophysiological adaptation of the blood–brain barrier (BBB) is not fully addressed in current therapeutic strategies, which may result in a lack of selective delivery to brain lesions [261]. This is especially relevant given the disruption of the BBB by astrocyte polarization towards a proinflammatory phenotype in neurodegenerative diseases, as well as the potential accumulation of oligonucleotides in specific tissues or organs [165,262]. Together, these factors can lead to unpredictable delivery and potential risks of aptamer-based therapy [263].

Last, aptamers are generally considered nonimmunogenic, but unintended activation of proinflammatory signalling or immunodeficiency may occur [153]. The immunostimulatory properties of unmethylated 20-deoxycytidine-phosphate-20-guanine (CpG) motifs, either alone or in longer DNA and RNA oligonucleotides, on the innate immune system, have long been reported [264]. These sequences can be found in viruses and bacteria that are recognized as PAMPs and further exaggerate immune responses via TLR9 [265]. CpG sequences can be potent molecular adjuvants that reinforce the immune response cascade, but they can also trigger unwanted immune responses [266,267]. Pharmaceutical reviews have addressed strategies such as excising the toxic CpG-containing segment to retain its essential binding site, modifying the backbone, and coadministering antagonistic or suppressive oligonucleotides [265]. Of note, the first FDA-approved aptamer, macugen, contains two CpG sequences, yet no serious adverse effects have been reported [268]. This is likely due to the localized route of administration (intraocular injection) and the molecular context of the aptamer, which hinders the activity of the CpG sequences [265]. On the other hand, immunodeficiency may arise from the consequence of ageing and the use of immunosuppressive drugs [269]. Thus, careful consideration of immunosuppressive drugs and their administration route is necessary when delivering therapeutics to the brain.

## 8. Conclusions and Future Perspectives

While aptamers hold promise as novel therapeutics for eliminating pathological proteins and regulating neuronal immune responses, their use in this context is still in the early stages of development. As with any new drug candidate, there is limited understanding of their optimal use, including dosage, administration route, and potential side effects. Furthermore, there are still some concerns about the viability of aptamers as neuronal immune regulators, including long-term usage and application to senescent cells. Future perspectives for the use of aptamers in the context of neuroinflammation in neurodegeneration should involve in-depth research into their pharmacokinetics and biodistribution.

Regarding the potentially adverse effects, including systemic toxicity and potential immunogenicity, it is necessary to carefully consider each case individually, accounting for factors such as the target, route of administration, and specific sequence of the aptamer. While successful examples such as Maceugen exist, there is no gold standard for assessing aptamers. Additionally, exploring their potential for targeted drug delivery, such as through the use of modified aptamers encapsulated in nanoparticles and targeted towards transferrin receptors (Apt-LP), as well as their use in combination therapies with existing treatments such as DOX-EpCAM-TfR and NXP032 (Vitamin C conjugated DNA Aptamin C320 complex), could offer promising avenues for clinical translation [270].

Aptamers can recognize a variety of targets without prior knowledge, even with subtle differences. As previously mentioned, many existing aptamers have been developed for numerous cytokines and cell surface receptors and have proven to be effective in in vivo neuroinflammation models. This presents a unique opportunity for the treatment of prolonged neuroinflammation, as there are currently limited effective treatment options available for NDDs. Future directions have been proposed recently by neuropathologists to address the implications of immunotherapy for NDDs [271]. These include (1) strengthening peripheral immunity to harness bone marrow-derived macrophages and regulatory T cells, (2) considering the brain–immune system as an ecosystem that targets whole-body anti-inflammation, rather than targeting the brain only, and (3) emphasizing the blockage of immune checkpoints involved in NDDs, such as PD-1 and its ligands, in the pathology of a dementia model.

Ongoing research should aim to deepen our understanding of the underlying mechanisms of neuroinflammation, such as the role of central immune cells and potential biomarkers. Clinical trials and studies are necessary to establish the clinical significance of these mechanisms and to develop aptamers for the effective diagnosis and treatment of NDDs and targeted drug delivery. Only through such research efforts can we advance the translation of aptamers from the laboratory bench to the bedside, eventually improving the quality of life of patients.

## Data Availability

Data sharing is not applicable to this article.

## Abbreviations

AD	Alzheimer’s Disease
ADME	Absorption, Distribution, Metabolism, and Excretion
AML	Acute Myeloid Leukaemia
APOE	Apolipoprotein E
APP	Amyloid Precursor Protein
BACE	Beta-Secretase
BBB	Blood–Brain Barrier
BMSC	Bone Marrow-Derived Mesenchymal Stem Cells
CD	Cluster of Differentiation
CME	Clathrin-Mediated Endocytosis
CNS	Central Nervous System
DAM	Disease-Associated Microglia
DAMP	Damage-Associated Molecular Pattern
DOX	Doxorubicin
ECM	Extracellular Matrix
EpCAM	Epithelial Cell Adhesion Molecule
LPS	Lipopolysaccharide
MAPK	Mitogen-Activated Protein Kinase
MRCC	Metastatic Renal Cell Carcinoma
MS	Multiple Sclerosis
MST1	Macrophage Stimulating kinase 1
MYD88	Myeloid Differentiation Primary Response 88
NDDs	Neurodegenerative Diseases
NSAIDs	Non-Steroidal Anti-Inflammatory Drugs
PAMP	Pathogen-Associated Molecular Pattern
PD	Parkinson’s Disease
PEG	Polyethylene Glycol
PML	Progressive Multifocal Leukoencephalopathy
POX	Paraoxon
PPAR	Peroxisome Proliferator-Activated Receptor
PRRs	Pattern Recognition Receptors
PS	Phosphatidylserine
ROS	Reactive Oxygen Species
RVG	Rabies Virus Glycoprotein
SELEX	Systematic Evolution of Ligands by Exponential Enrichment
TBI	Traumatic Brain Injury
TLR	Toll-Like Receptor
TREM	Triggering Receptor Expressed on Myeloid Cells
